# Neurometabolic characteristics in the anterior cingulate gyrus of Alzheimer’s disease patients with depression: a ^1^H magnetic resonance spectroscopy study

**DOI:** 10.1186/s12888-015-0691-7

**Published:** 2015-12-02

**Authors:** Zhongwei Guo, Jiangtao Zhang, Xiaozheng Liu, Hongtao Hou, Yulin Cao, Fuquan Wei, Japeng Li, Xingli Chen, Yuedi Shen, Wei Chen

**Affiliations:** Tongde Hospital of Zhejiang Province and Zhejiang Mental Health Center, Hangzhou, Zhejiang 310012 China; Center for Cognitive Brain Disorders and Zhejiang Key Laboratory for Research in Assessment of Cognitive Impairments, Hangzhou Normal University, Hangzhou, Zhejiang 310015 China; The Affiliated Hospital of Hangzhou Normal University, Hangzhou, Zhejiang 310015 China; Department of Psychiatry, Sir Run Run Shaw Hospital, Zhejiang University School of Medicine, and the Collaborative Innovation Center for Brain Science, No. 3 East Qingchun Road, Hangzhou, Zhejiang 310016 China; Key Laboratory of Medical Neurobiology of Chinese Ministry of Health, Hangzhou, Zhejiang 310016 China

**Keywords:** Alzheimer’s disease, Depression, Magnetic resonance spectroscopy, Anterior cingulate gyrus

## Abstract

**Background:**

Depression is a common comorbid psychiatric symptom in patients with Alzheimer’s disease (AD), and the prevalence of depression is higher among people with AD compared with healthy older adults. Comorbid depression in AD may increase the risk of cognitive decline, impair patients’ function, and reduce their quality of life. However, the mechanisms of depression in AD remain unclear. Here, our aim was to identify neurometabolic characteristics in the brain that are associated with depression in patients with mild AD.

**Methods:**

Thirty-seven patients were evaluated using the Neuropsychiatric Inventory (NPI) and Hamilton Depression Rating Scale (HAMD-17), and divided into two groups: 17 AD patients with depression (D-AD) and 20 non-depressed AD patients (nD-AD). Using proton magnetic resonance spectroscopy, we characterized neurometabolites in the anterior cingulate gyrus (ACG) of D-AD and nD-AD patients.

**Results:**

Compared with nD-AD patients, D-AD patients showed lower N-acetylaspartate/creatine (NAA/Cr) and higher myo-inositol/creatine (mI/Cr) in the left ACG. NPI score correlated with NAA/Cr and mI/Cr in the left ACG, while HAMD correlated with NAA/Cr.

**Conclusions:**

Our findings show neurometabolic alterations in D-AD patients. Thus, D-AD pathogenesis may be attributed to abnormal activity of neurons and glial cells in the left ACG.

## Background

Depression is a common comorbid psychiatric symptom in patients with Alzheimer’s disease (AD). It is associated with cognitive decline in AD patients, and reduced quality of life in patients and their caregivers [1]. Currently, there are no effective therapeutic interventions. Therefore, discovering and understanding the mechanisms and biological signatures related to depression in AD has clinical value.

Neuroimaging studies have yielded mixed results on regional brain volume differences between depressed and non-depressed AD patients. Son et al. [2] reported that AD patients with depression show decreased gray matter volume in the left inferior temporal gyrus compared with non-depressed AD patients. Moreover, Lebedev et al. [3] reported cortical thinning in left parietal and temporal brain regions in AD patients with depressive symptoms compared with non-depressed AD patients. They also reported strong negative correlation between cortical thickness in the precuneus and parahippocampal cortex and total tau (t-τ) (an AD biomarker) in the cerebrospinal fluid of depressed compared with non-depressed AD patients. Hu et al. [4] reported significant correlation between depression assessed with the Neuropsychiatric Inventory (NPI) and gray matter atrophy in the left middle frontal cortex. However, this was not supported by Bruen et al. [5]. Studies using single photon emission computed tomography show reduced perfusion in the middle frontal gyrus [6] and dorsolateral prefrontal (DLPFC) [7] regions in AD patients with depression.

These conflicting findings may partly be owing to limitations of the image analysis methods. For example, metabolic data from positron emission tomography (PET) studies are usually expressed relative to the whole brain or a specific structure. Therefore, as it does not reflect an absolute metabolic rate, PET may only indirectly reflect regional neuronal activity. In addition, structural measures are not sensitive to neuronal function at early stages [8]. Histological studies of postmortem samples from well-characterized depressed individuals who have committed suicide show altered cortical dendritic branching of pyramidal neurons in the ACC [9]. Additionally, the ratio of primed over ramified microglia in the dorsal ACC is significantly increased in depressed individuals who have committed suicide compared with healthy controls [10]. Hence, investigation of neurons and glial cells may aid our understanding of depression pathogenesis in AD patients.

Proton magnetic resonance spectroscopy (^1^H-MRS) is a noninvasive magnetic resonance imaging (MRI) method to measure brain metabolite concentrations, including the neuronal marker N-acetylaspartate (NAA), the membrane phospholipid product choline (Cho), the second messenger metabolite and gliosis marker myo-inositol (mI), and total creatine (Cr), which includes creatine and phosphocreatine and is used as an internal standard [11]. ^1^H-MRS has been used in clinical studies of major depressive disorder (MDD) [12, 13] and AD [11]. Studies in patients with MDD compared with healthy controls report decreased NAA [14], decreased mI [15], and normal Cho/Cr [16] in the anterior cingulate cortex (ACC). Furthermore, treatment of MDD with lamotrigine and antidepressants may increase NAA and mI in the ACC [14, 15]. ^1^H-MRS findings also suggest that AD patients exhibit reduced NAA/Cr and elevated mI/Cr ratios in the medial temporal lobe, posterior cingulate gyrus, temporoparietal lobe, hippocampus, and prefrontal lobe [11].

Recently, several studies have been performed using ^1^H-MRS in AD patients with behavioral and psychological symptoms. One study of 30 AD patients found significantly lower NAA/Cr and higher mI/Cr ratios in patients with delusions compared to those without. Additionally, patients with activity disturbances had significantly lower NAA/Cr in the ACC compared to those without, but there was no relationship between depression and NAA/Cr or mI/Cr [17]. Another study of 36 AD patients, 19 patients with amnestic mild cognitive impairment (aMCI), and 23 cognitively normal (CN) subjects revealed statistically significant correlations between mI/Cr in ACG and total NPI scores. However, the relationship between the depressive domain of NPI and brain metabolites was not specifically investigated [18]. Tsai et al. [19] reported a positive correlation between depression in AD and the Cho/Cr ratio in the left DLPFC, and mI/Cr ratio in both the left and right cingulate gyrus. In contrast, in a trial of donepezil versus memantine treatment for AD, no significant correlations between ^1^H-MRS in the ACG and depression were reported [20]. Therefore, the underlying pathophysiology of AD with depression remains unclear. Inconsistencies may result from heterogeneity in previous research subjects, for example, AD patients may have more than one component of behavioral and psychological symptoms. Furthermore, few studies of depression in AD patients using ^1^H-MRS have been performed to date.

The main purpose of our study was to investigate the relationship between depression and brain metabolites in AD patients. Given the known involvement of the ACG in MDD and AD, we examined its contribution to depression in AD patients, and hypothesized that metabolic changes would be observed in depressed AD patients. Thus, we investigated ACG metabolites using ^1^H-MRS in AD patients with and without depression.

## Methods

### Patients

Thirty-seven patients with mild AD were recruited between December 2013 and December 2014 from Zhejiang Mental Health Center, China. All patients met the criteria for probable AD from the National Institute of Neurological and Communicative Diseases and Stroke and Alzheimer’s Disease and Related Disorders Association [21]. Patients’ scores ranged from 18 to 24 on the Mini-Mental State Examination (MMSE) and all patients had a score of 1 on the Clinical Dementia Rating (CDR) scale. A diagnosis of depression was confirmed using the *Diagnostic and Statistical Manual of Mental Disorders*, fourth edition (DSM-IV) [22]. Depression severity was assessed using the Hamilton Depression Rating Scale (HAMD-17) and NPI [23]. AD patients with depression (D-AD) had a score of 1 on the depression domain and a score of 0 on any of the other 11 NPI domains, and scored ≥ 7 on HAMD-17. Based on recommendations for inclusion criteria for clinical trials, D-NPI scores of ≥ 4 are indicative of clinical significance [24]. Non-depressed AD patients (nD-AD) did not meet DSM-IV criteria for depression. All scales were administered by trained neuropsychologists. All patients were right-handed, had more than 6 years’ education, and were aged between 65 and 80 years. Caregivers were a patient’s spouse and/or first-degree relative, and also had more than 6 years’ education.

Patients with a history of neurological disorders (e.g., active epilepsy), psychiatric illnesses (e.g., schizophrenia, major depression, or mania), traumatic brain injury, those taking psychotropic medications, and significant alcohol and/or other substance abuse issues were excluded. To minimize the risk of concomitant vascular pathology, subjects were also excluded if dual-echo MR images showed two or more hyperintense lesions with diameters ≥ 5 mm, or more than four hyperintense lesions with diameters between 0 and 5 mm.

All participants (or their legal representatives) provided formal written consent. The research protocol was approved by the Ethics Committee of Tongde Hospital of Zhejiang Province (No.2013-001).

### MRI and MR spectroscopy

**Fig. 1:**
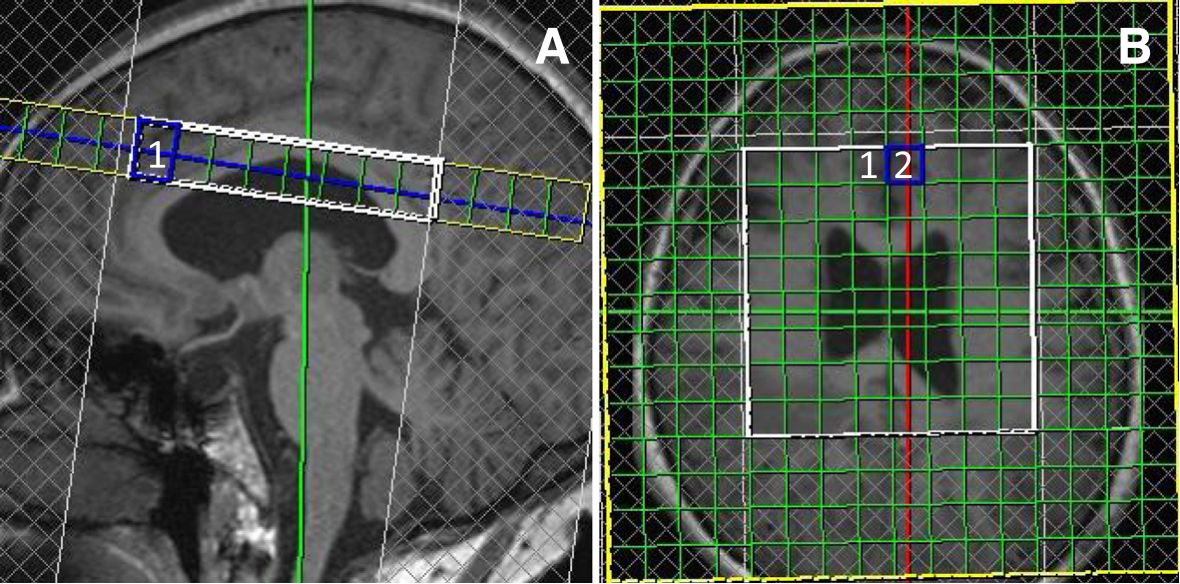
Axial T2-weighted localizing images for placement of the MRS region-of-interest. The voxel for MRS examination was located in the left anterior cingulate gyrus (ACG) (rectangle 1) at the mid-sagittal level (**a**), and right ACG (square 1) and left ACG (square 2) in the coronal section (**b**). Voxel size = 10 mm × 10 mm × 15 mm.

MRI and ^1^H-MRS were acquired for all study participants on a 3.0 Tesla unit (Siemens MAGNETOM Verio; Siemens Medical Systems, Erlangen, Germany) using an eight-channel phased-array head coil. Foam padding and headphones were used to reduce head motion and scanner noise. Advanced shimming, as provided by the manufacturer, was performed automatically to optimize field homogeneity. A two-dimensional chemical shift imaging sequence (CSI), using a point-resolved spectroscopy (PRESS) technique, was used to acquire water-suppressed ^1^H-MRS simultaneously from the left and right cingulate region. The imaging parameters were: echo time (TE) = 35 ms, repetition time (TR) = 1500 ms, and average = 4, including ‘weighted’ mode used for k-space acquisition, with matrix size = 16 × 16 without interpolation, field-of view (FOV) = 160 mm, voxel of interest (VOI) = 80 mm, slice thickness = 1.5 cm, and ‘fully excited VOI’ was switched on. T2-weighted transverse, sagittal, and coronal gradient echo images (TR/TE = 600/95 ms) were acquired to localize the ^1^H-MRS signal. A line was drawn perpendicular to the AC-PC line, which also cut across the medial section of the genu of the corpus callosum. The VOI in the anterior cingulate was located in front of the line. The VOI mainly consisted of the dorsal ACC and part of the lateral prefrontal cortex (PFC) [25, 26] (Figure 1).

**Fig. 2:**
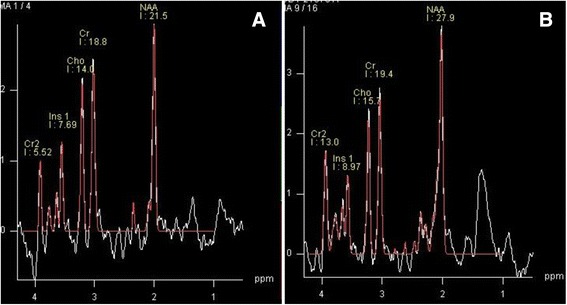
Examples of ^1^H-MRS spectra acquired from one voxel in D-AD and AD groups. Note the lower NAA/Cr and higher mI/Cr ratios in D-AD subjects (**a**) compared with nD-AD (**b**) (CSI, PRESS, voxel size = 10 mm × 10 mm × 15 mm).

Total acquisition time for both MRI and MRS was approximately 10 min. Spectra data were post-processed using a commercially available, spectral analysis software package (Syngo spectroscopy post-processing package, Siemens Healthcare, Erlangen, Germany). The spectrum covered a frequency range of 4.3–0.1 ppm. Peak areas of NAA, mI, and Cr were estimated, and ratios of the area under each peak were expressed relative to Cr in each spectrum (Figure 2). Voxel placement for spectroscopy and all data analysis were performed by one experienced radiologist who was blind to each subjects’ diagnosis, and confirmed by another radiologist and one physician. VOI position was carefully adjusted to minimize white matter and cerebrospinal fluid contamination.

### Data analysis

Statistical analyses were performed using the Statistical Package for the Social Sciences, Version 15.0 (SPSS, Inc., Chicago, IL, USA). Demographic and clinical characteristics of D-AD and nD-AD patients were assessed using independent-samples *t*-tests and χ^2^ tests. Differences between metabolite ratios relative to Cr (NAA/Cr, Cho/Cr, and mI/Cr) in the left and right ACG were tested using independent-samples *t*-tests. Pearson’s correlation analysis was used to investigate the correlation between metabolite ratios and NPI and HAMD scores. Statistical significance was set at *P* < 0.05.

## Results

Table 1 lists the demographic and clinical data for both groups. Thirty-seven mild AD patients were included in the study. Seventeen AD patients had depressive symptoms. Scores on the depression domain of NPI (D-NPI) ranged between 4 and 9, with a mean of 6.76 ± 2.1. There were no significant differences in sex, age, education or MMSE between the D-AD and nD-AD groups (*P* > 0.05).

**Table 1:** Demographics and neuropsychological data

	D-AD	nD-AD	t/χ^2^	*p*
Gender, n (M/F)	17(8/9)	20(9/11)	0.016	0.900
Age, years	72.9 ± 5.5	74.7 ± 6.3	−0.892	0.379
Education, years	8.6 ± 1.84	8.5 ± 1.83	0.213	0.853
MMSE	21.6 ± 2.8	22.1 ± 2.4	−0.603	0.551
D-NPI	6.76 ± 2.1	0	-	-
HAMD	12.3 ± 3.85	3.8 ± 1.57	14.150	0.000

Data represent mean ± SD. Data were analyzed using independent-samples *t*-tests and χ^2^ tests

*AD* alzheimer’s disease, *D-AD* alzheimer’s disease with depression, *nD-AD* non-depressed AD patients, *M* male, *F* female, *MMSE* mini-mental state examination, *D-NPI* depression domain of neuropsychiatric inventory, *HAMD* hamilton depression rating scale

Table 2 shows the ratio of measurements obtained by ^1^H-MRS. There were significant differences in the ratios of NAA/Cr and mI/Cr in the left ACG. In addition, the D-AD group had a lower NAA/Cr ratio than the nD-AD group (1.35 ± 0.18 *vs.* 1.50 ± 0.23, respectively; *t* = −2.161, *P* < 0.05). Similarly, the D-AD group had a higher mI/Cr ratio than the nD-AD group (0.66 ± 0.13 *vs.* 0.58 ± 0.09, respectively; *t* = 2.213, *P* < 0.05). No differences were found in ratios between groups in the right ACG (*P* > 0.05).

**Table 2:** ^1^H-MRS in the left and right ACG between D-AD and nD-AD groups

		D-AD	nD-AD	t	*p*
	NAA/Cr	1.35 ± 0.18	1.50 ± 0.23	−2.161	0.038
Left ACG	Cho/Cr	0.94 ± 0.25	0.89 ± 0.14	0.846	0.403
	mI /Cr	0.66 ± 0.13	0.58 ± 0.09	2.213	0.033
	NAA/Cr	1.47 ± 0.28	1.52 ± 0.40	−0.463	0.646
Right ACG	Cho/Cr	0.89 ± 0.18	0.87 ± 0.12	0.463	0.646
	mI /Cr	0.61 ± 0.13	0.59 ± 0.09	0.305	0.762

Data represent mean ± SD

*ACG* anterior cingulate gyrus, *AD* alzheimer’s disease, *D-AD* alzheimer’s disease with depression, *nD-AD* non-depressed AD patients

Pearson’s correlation analysis of the D-AD group detected statistically significant correlations between NPI scores and NAA/Cr and mI/Cr ratios of the left ACG (*r* = −0.717, *P* = 0.001; *r* = 0.492, *P* = 0.045). Similarly, statistically significant correlation between HAMD and NAA/Cr ratio of the left ACG was detected (*r* = −0.778, *P* = 0.000).

## Discussion

To the best of our knowledge, this is the first study to examine neurometabolic characteristics by ^1^H-MRS in the ACG of AD patients with depression. The major findings of our study are: (1) Compared with nD-AD patients, D-AD patients show lower NAA/Cr and higher mI/Cr ratios in the left ACG; and (2) correlation between NPI and HAMD scores and NAA/Cr and mI/Cr ratios of the left ACG suggest abnormalities in ACG neurometabolites are associated with depression in AD.

The ACG processes the integration of cognition and affect [27, 28]. It is also implicated in MDD, in which abnormal metabolism [29, 30], decreased blood flow [31], reduced gray matter volume [32–34], and ACG involvement in disrupted brain networks [35] have been observed. In a post-mortem examination of depressed individuals that committed suicide, alterations in dendritic branching and microglial phenotypes were observed in the ACC [9]. A recent neuroimaging study of depression in AD found that depression is associated with damage to structures in specific neural networks and functional disruption of cortical neural systems involving the ACG [8]. Therefore, the ACG is considered a crucial region in the neuronal circuitry underlying depression pathophysiology. Our observation that D-AD patients have abnormalities in ACG neurometabolites is consistent with this. Accordingly, it is likely that the ACG also underlies depressive symptoms in AD patients.

NAA is the most prominent ^1^H-MRS peak, and only found in the nervous system. It is a marker of neuronal density or function, osmoregulation, and energy homeostasis. There is a direct relationship between NAA synthesis, oxygen consumption, and ATP production in the central nervous system [36, 37]. Reduction in NAA levels measured by ^1^H-MRS are a recognized marker of neuronal loss or dysfunction in depressive disorders [38]. Previous studies have demonstrated that MDD and bipolar disorder patients have lower NAA/Cr levels than healthy controls in the PFC, medial frontal cortex, and ACG [14, 15, 39, 40]. Longitudinal research also shows that NAA/Cr in the pregenual ACC of patients with MDD significantly decreases over 9–10 months, and at baseline, has a logarithmic negative association with illness duration [41]. Furthermore, successful treatment of MDD with antidepressants is associated with normalization of NAA levels in the ACC [14, 15]. More specifically, a growing body of research suggests that MDD patients exhibit decreased beta nucleoside triphosphates compared with healthy controls [42, 43]. These studies, along with our finding of lower NAA/Cr in D-AD patients, implicates neuronal degeneration and dysfunction in the ACG of depressed AD patients.

mI is considered a maker of glial proliferation [44]. It is also involved in the regulation of neuronal osmolarity, metabolism of membrane-bound phospholipids, and the phosphoinositide secondary messenger pathway. Several animal studies of depression report higher mI/Cr in the PFC of depressed compared with control animals [45, 46]. Lirng et al. [47] observed that migraine patients with MDD had higher mI/Cr ratios in the bilateral DLPFC compared with patients without MDD. Furthermore, mI/Cr in the right DLPFC positively correlates with scores on the Beck Depression Inventory, suggesting that increased mI/Cr within the DLPFC might be associated with MDD in migraine patients. Torres-Platas et al. [48] reported hypertrophic astrocytes in the ACG of 10 well-characterized depressed suicide cases. Recently, they reported increased microglial priming and increased gene expression of microglial markers in the dorsal ACG in postmortem brain samples from middle-aged depressed individuals who committed suicide [10]. Higher mI/Cr in the ACG of D-AD patients observed in our study is consistent with these previous studies. We suggest that higher mI/Cr in the ACG region reflects increased glial content and activation. Therefore, our study provides further evidence for the involvement of ACG glial cells in depression of AD patients.

We also found that D-AD patients have abnormal neurometabolic changes in the left ACG, but not the right ACG, suggesting asymmetrical alterations across hemispheres. Prior studies have also shown evidence of asymmetrical ACG abnormalities in late-life depressed (LLD) patients. Disabato et al. [49] found significantly smaller left anterior cingulate thickness in late-onset LLD compared with early-onset LLD subjects. The late-onset group also had more hyperintensities than early-onset LLD subjects. Similarly, Yuan et al. [50] found abnormal left ACG volume in geriatric depression (RGD) patients compared with healthy control subjects. Furthermore, there was a significant correlation between left ACG volume and Rey Auditory Verbal Learning Test delayed recall raw score in RGD patients. Ritchie et al. [51] confirmed that early-onset depression and late-onset depression exhibit heterogeneity in etiology, including onset age. These findings indicate that age at onset of depressive symptoms in LLD subjects is associated with differences in cortical thickness. Moreover, the left ACG might be involved in psychopathology and pathophysiology of RGD. One study using ^11^C-Pittsburgh Compound B PET imaging found more amyloid plaques in the ACG of LLD patients compared with controls [52]. Meanwhile, another longitudinal study of brain metabolic changes during the conversion from aMCI to AD, found that converters had a significantly greater metabolic decrease in the left ACG than non-converters [53]. Therefore, we speculate that the left ACG might be involved in the psychopathology of depression in AD patients.

Some potential limitations of our study should be taken into consideration. First, we used a semi-quantitative MRS approach, in which the intensity of each metabolite is normalized to Cr under the assumption that Cr concentration remains relatively constant in different brain diseases. This method is the most frequently used method for clinical MRS studies [54]. However, variations in Cr concentration are present during tissue destruction or in systemic diseases. Subsequent studies with absolute measures may revalidate our findings. Second, only the ACG was investigated. Therefore, even though neurodegenerative processes are suggested in D-AD, caution should be taken to extrapolate the findings to other brain areas. Third, although most demographic or clinical features were relatively balanced between both groups, the sample size was rather small. Thus, these preliminary results need further replication with a larger sample size.

## Conclusions

We found decreased NAA/Cr and increased mI/Cr ratios in the left ACG of AD patients with depression that correlated with NPI and HAMD scores. This suggests that neural and glial dysfunction in the left ACG is associated with psychopathology of depression in AD patients. As such, neurometabolic changes in the left ACG could be a potential “biological signature” of depression related to AD, and a target for novel treatments for AD patients with depression.

## Abbreviations

AD: Alzheimer’s disease
MCI: Mild cognitive impairment
ACG: Anterior cingulate gyrus
PCG: Posterior cingulate gyrus
